# Multidisciplinary Integrated Metabolic Rehabilitation in Elderly Obese Patients: Effects on Cardiovascular Risk Factors, Fatigue and Muscle Performance

**DOI:** 10.3390/nu11061240

**Published:** 2019-05-31

**Authors:** Antonello E. Rigamonti, Alessandra De Col, Sofia Tamini, Sabrina Cicolini, Diana Caroli, Roberta De Micheli, Gabriella Tringali, Laura Abbruzzese, Nicoletta Marazzi, Silvano G. Cella, Alessandro Sartorio

**Affiliations:** 1 Department of Clinical Sciences and Community Health, University of Milan, via Vanvitelli 32, 20129 Milan, Italy; silvano.cella@unimi.it; 2 Istituto Auxologico Italiano, IRCCS, Experimental Laboratory for Auxo-Endocrinological Research, strada L. Cadorna 90, 28824 Piancavallo (VB), Italy; a.decol@auxologico.it (A.D.C.); s.tamini@auxologico.it (S.T.); s.cicolini@auxologico.it (S.C.); d.caroli@auxologico.it (D.C.); r.demicheli@auxologico.it (R.D.M.); g.tringali@auxologico.it (G.T.); l.abbruzzese@auxologico.it (L.A.); n.marazzi@auxologico.it (N.M.); sartorio@auxologico.it (A.S.); 3 Istituto Auxologico Italiano, IRCCS, Division of Metabolic Diseases, strada L. Cadorna 90, 28824 Piancavallo (VB), Italy

**Keywords:** multidisciplinary integrated metabolic rehabilitation, diet, exercise, geriatric obesity

## Abstract

Background: Obesity is a widespread problem in the elderly, being associated with severe comorbidities negatively influencing life expectancy. Integrated multidisciplinary metabolic rehabilitation aimed to reduce body weight (BW) and fatigue, increase physical autonomy and introduce healthy life style changes has been proposed as a useful intervention to improve the general health status and quality of life of the obese geriatric population. Methods: Six hundred-eighty four severely obese subjects (F/M = 592/92; age range: 61–83 years; mean body mass index, BMI ± SD: 42.6 ± 5.6 kg/m^2^) were admitted to take part in a three-week in-hospital BW reduction program (BWRP), entailing energy restricted diet, psychological counselling, physical rehabilitation and nutritional education. Biochemical parameters, cardiovascular risk factors (throughout the Coronary Heart Disease Risk, CHD-R), fatigue (throughout the Fatigue Severity Scale, FSS) and lower limb muscle performance (throughout the Stair Climbing Test, SCT) were evaluated before and at the end of the BWRP. Results: A 4% BW reduction was achieved at the end of the BWRP. This finding was associated with a significant improvement of the metabolic homeostasis (i.e., decrease in total cholesterol and glucose) and a reduction of systolic blood pressure in both females and males, thus resulting in a reduction of CHD-R in the male group. Total FSS score and SCT time decreased in female and male obese patients. The effects of BWPR were comparable among all age-related subgroups (>60, 60–69 and >70 years), apart from ΔCHD-R, which was higher in male subgroups. Finally, age was negatively correlated with ΔBMI and ΔFSS. Conclusions: Though only a relatively limited number of outcomes were investigated, the present study shows that a 4% BW reduction in severely elderly obese patients is associated with positive multisystemic effects, particularly, muscle-skeletal and cardiometabolic benefits, which can favorably influence their general well-being and improve the autonomy level in performing more common daily activities. The maintenance of a healthy life style, including controlled food intake and regular physical activity, after a BWRP is obviously recommended in all elderly obese patients to further improve their clinical condition.

## 1. Introduction

In the last decade, we have assisted a dramatic worldwide increase in geriatric population and, concomitantly, in obesity [1]. Thus, the elderly obese patient is becoming an increasingly prevalent phenotype in the general population from developed and also developing countries, with relevant socioeconomic implications for the public health system and political decision-making [2].

Geriatric obesity is associated with a worsening of the coronary heart disease risk (CHD-R), due to dyslipidemia, hypertension, type 2 diabetes mellitus and physical inactivity [3]. Furthermore, functional autonomy and, in general, the quality of life are consistently reduced in an elderly obese subject, so that obesity can be considered a determinant of “frailty” in geriatric practice and have a prominent causative role for several clinical conditions that require hospitalization or institutionalization [4]. Finally, elderly women seem to be at high risk to develop or maintain a pre-existing obesity due the well-known post-menopausal changes [5].

Body weight (BW) reduction programs (BWRPs) have been demonstrated to be a valid strategy to contrast the negative effects associated with geriatric obesity, particularly when a multidisciplinary integrated approach is adopted. In this context, diet combined with physical activity and psychological counseling has been shown to be more effective when administered in cohorts of elderly obese patients in comparison with the single interventions [6].

Nevertheless, some controversies regarding the short- and long-term benefits and safety of BWRPs against geriatric obesity still persist [4].

For instance, elderly subjects have been reported to have lower resting energy expenditure and caloric requirements, with the consequence that a BWRP may induce an unsatisfactory negative energy balance and a negligible weight loss [7,8,9,10].

In addition, since sarcopenic obesity is more prevalent in elderly than young individuals, a protein-restricted dietetic regimen administered to an elderly obese patient could increase the catabolism of muscle proteins, a process amplified by the obesity-associated low-grade chronic inflammation and aging-related hypomotility [11,12]. Weight loss obtained in an elderly obese patient undergoing caloric restriction may derive from a relevant shrinkage of lean mass (more than 25%), with possible reduction in muscle strength, limitations in motor function and impairment of glucose tolerance, with muscle tissue being fundamental for glucose uptake and glucometabolic homeostasis [13,14,15,16,17].

Independently from age, weight loss reduces bone mass density, an effect that, in osteopenic or osteoporotic post-menopausal women, could be deleterious due the risk of fractures [18].

Finally, some authors have proposed the “obesity paradox” to indicate the protective value of a high BMI in later life [19]. In particular, the progression of some clinical conditions, frequently diagnosed in geriatric patients, such as cancer, chronic heart failure and end-stage renal disease, is slowed down to wasting syndrome or cachexia in the obese subgroup compared to the lean counterpart [20].

Based on these conflicting results, well highlightened by recent reviews [4,6], there is the urgent need to evaluate adjunctive outcomes in order to define and quantify the effects of an (integrated) BWRP on cardiometabolic status, muscle performance and quality of life in elderly obese patients. Understanding of the beneficial effects of any BWRP on specific outcomes in different subgroups of elderly obese patients (e.g., for gender and age ranges) might allow us to define the demographic and clinical characteristics of the elderly obese patient who will favorably respond to a standardized BWRP and, additionally, to tailor each component of the BWRP (dietetic regimen, type/duration/intensity of physical activity, adherence to healthy life styles, concomitant pharmacological therapy etc.) to a particular elderly obese patient having specific demographic and clinical characteristics.

Therefore, the present study was aimed at investigating the effects of a three-week BWRP, administered to a large cohort of elderly obese females and males, on (1) CHD-R, a validated scoring system, including demographic, clinical, biochemical and cardiovascular parameters, which permits the calculation of CHD-R over the next 10 years and compare this value to that of others of the same age [21]; (2) stair climbing test (SCT), used to evaluate functional strength, balance and agility of lower limbs through ascending a set number of steps [22,23]; and (3) fatigue severity scale (FSS), a largely employed self-report questionnaires to evaluate fatigue in daily activities, which does not depend upon an underlying depressive condition [24].

## 2. Material and Methods

### 2.1. Patients and Body Weight Reduction Program

**Figure 1:**
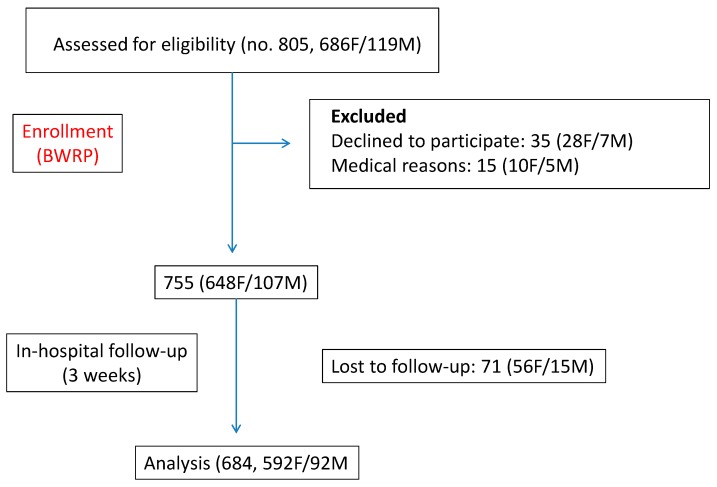
Consort flow diagram of the study. BWRP, body weight reduction program.

Six-hundred-eighty-four severely obese subjects (females, F/males, M = 592/92; age range: 61–83 years; body mass index, BMI: 42.6 ± 5.6 kg/m^2^) were recruited at the Division of Metabolic Diseases, Istituto Auxologico Italiano, Piancavallo (VB), where they were hospitalized for a three-week multidisciplinary integrated BWRP, including hypocaloric diet, nutritional education, psychological counselling and moderate physical activity (see below for details). The sample size was considered adequate, taking into account a power analysis in which a mean value of ΔBMI (%) after BWRP was supposed to be equal to 4.0 ± 4.0% with an α error of 0.05 at two tails and a power of 0.80. The consort flow diagram is shown in Figure 1. The unique criterion of inclusion was a BMI > 35 kg/m^2^, while the main exclusion criteria were physical inability in performing SCT and cognitive impairments hampering the FSS execution.

When considering the BWRP in detail, energy intake was restricted by imposing a diet (5023–7113 kJ/day, i.e., 1200–1700 kcal/day) containing about 21% proteins, 53% carbohydrates and 26% lipids. The calories to be given with the diet were calculated by subtracting approximately 25% from the value of resting energy expenditure as measured in each patient by indirect calorimetry (Vmax 29; SensorMedics Corporation, Yorba Linda, CA, USA) for a total duration of 20 min. Under the energy restriction, each patient was free to choose foods from a heterogeneous daily menu. Foods to which the patient declared to be allergic were removed from the menu. Five daily portions of fruits and vegetables were obligatory. A fluid intake of at least 1500 mL/day was encouraged. Nutritional education consisted of lectures, demonstrations and group discussions with and without a supervisor, took place every day throughout the whole rehabilitation period. Sessions of psychological counselling were conducted by clinical psychologists 2–3 times/week and were based on individual or cognitive behavioral strategies. Physical activity consisted of five training sessions/week (about 1 h each session), including indoor light jogging, dynamic exercises of the upper and lower limbs (standing and floor gymnastics routines, focalized on muscle strength-power development) at moderate intensity under the guide of a therapist; furthermore, subjects underwent either 15–20 min aerobic exercise or 2 km outdoor walking on a predetermined track, according to individual capabilities and clinical status.

Before and after BWRP, each subject underwent the following tests/evaluations, which are described in detail below:

–SCT (stair climbing test);

–FSS (fatigue severity score);

–CHD-R (coronary heart disease risk);

–Standard hematochemistry.

The protocol was approved by the local Ethical Committee (research project code: 18A301, acronym: FUOBAUXO); all subjects gave their written consent after being fully informed of the nature and procedures of the study.

### 2.2. Stair Climbing Test

SCT is a well-standardized procedure, which was readapted and validated by our group to measure the maximal anaerobic power [22,23,25]. Subjects were invited to climb up ordinary stairs (13 steps of 15.3 cm each, vertical distance: 1.99 mt) at the highest possible speed, according to their capabilities. The stairs were climbed one time by the participant. The time taken to perform the test was measured by an experimental investigator with a digital stopwatch. In line with the assumptions by Margaria et al. [26], anaerobic power (in W) was calculated by using the following formula:

(body weight × 9:81 × vertical distance)/time

where body weight, vertical distance and time are expressed in kg, m and sec, respectively, and 9.81 m/sec^2^ represents the acceleration of gravity. See also Reference [27] for further details.

### 2.3. Fatigue Severity Scale

FSS is one of the most commonly used self-report questionnaires for the fatigue assessment in chronic diseases [28,29], already used and validated in Italian obese patients by our group [24].

FSS consists of nine statements (items) describing the negative effects of fatigue on motivation, exercise, physical functioning, ability to carry out duties, work, family or social life. Responders are asked to rate each statement considering the previous week and using a Likert scale ranging from one (strong disagreement) to seven (strong agreement). The total score is computed by averaging the raw scores of each item.

### 2.4. Evaluation of Coronary Heart Disease Risk

Selected CHD-R factors, including systolic and diastolic blood pressures (SBP and DBP, respectively), total and high-density lipoprotein cholesterol (T-C and HDL-C, respectively), cigarette smoking and diabetes mellitus, were assessed in each subject [30]. Two BP determinations were made after the participant had been sitting at least 5 min, being the mean value used for the subsequent analyses.

BP and T-C/HDL-C levels were considered without regard to the use of anti-hypertensive or lipid-lowering drugs. The diagnosis of diabetes mellitus was defined if the patient was under treatment with insulin or oral hypoglycemic agents or, alternatively, if fasting blood glucose levels exceeded 140 mg/dL. Individuals who smoked at least one cigarette per day during the previous 12 months were classified as smokers.

The CHD-R scores were estimated using a simple CHD prediction model developed by Wilson et al. [30], which takes into account gender, age, diabetes, smoking, BP, T-C and HDL-C categories.

This scoring scale, based on established, independent and biologically important risk factors for CHD, represents a simplified approach to predict risk for initial CHD events in disease-free outpatients at 10 years.

CHD score sheets (for men and women) attribute different ranks in the function of classes of age (nine subgroups, from 30 up to 74 years), T-C (five subgroups, from <160 to ≥280 mg/dL), HDL-C (five subgroups, from <35 up to ≥60 mg/dL) and BP (SBP: Five subgroups, from <120 up to ≥160 mmHg; DBP: Five subgroups, from <80 up to ≥100 mmHg). Diabetes and smoking were defined into two categories (yes/no).

When SBP and DBP fell into different categories, the higher category was selected for the calculation of the CHD score.

### 2.5. Hematochemical Testing

Serum glucose level was measured by the glucose oxidase enzymatic method (Roche Diagnostics, Monza, Italy). The sensitivity of the method was 2 mg/dL.

Colorimetric enzymatic-assays (Roche Diagnostics, Monza, Italy) were used to determine serum T-C and HDL-C levels. The sensitivities of the assays were 3.86 mg/dL and 3.09 mg/dL, respectively.

### 2.6. Statistical Analysis

The Sigma Stat 3.5 statistical software package (Systat Software, San Jose, CA, USA) was used for data analyses and the GraphPad Prisma 5.0 software (GraphPad Software, San Diego, CA, USA) for data plotting.

The Shapiro-Wilk test showed that all parameters were normally distributed.

Results are reported as mean ± SD (standard deviation). Each parameter, particularly BW, BMI, T-C, HDL-C, glucose, SBP, DBP, CHD-R, FSS score and SCT time, were evaluated not only as an absolute value, but also as a pre-post-BWRP difference (Δ in the corresponding unit of measurement for CHD-R or % for all remaining parameters).

All parameters were compared among all subgroups (all, females/males, >60 years, 60–69 years and >70 years) before and after BWRP by using a t-Student test (for paired or unpaired data) or one-way ANOVA, followed by the post hoc Tukey’s test, if appropriate. A linear regression was applied to correlate each other parameter.

A level of significance of *p* < 0.05 was used for all data analyses.

## 3. Results

As shown in Table 1, Table 2 and Table 3, WHR, height, BW and BMI were significantly higher in males than females for each age range (>60 years, 60–69 years and >70 years) (*p* < 0.01) before BWRP, while significantly lower HDL-C levels were found in males than females (*p* < 0.01). The CHD-R, FSS score and SCT time were significantly higher in females than males (*p* < 0.01), with the exception of the subgroups aged >70 years. Males or females aged >70 years had a significantly lower BW when compared to the other subgroups of the same gender (i.e., >60 years and 60–69 years) (*p* < 0.01). BMI was significantly higher in females aged 60–69 years than in those aged >70 years (*p* < 0.01). Females aged >70 years had a significantly higher SCT time than those belonging to the subgroup of the same gender, aged 60–69 years (*p* < 0.01). When pooling BW or BMI values of females and males (i.e., total), subjects aged >70 years had a significant lower BW or BMI than the remaining subgroups (i.e., >60 years and 60–69 years) (*p* < 0.01). When pooling HDL-C levels of females and males (i.e., total), subjects aged >70 years had significant lower HDL-C levels than the subgroup aged 60–69 years (*p* < 0.01). CHD-R was significantly higher in subjects aged 60–69 years than in those aged >70 years, including both females and males (*p* < 0.01).

**Figure 2:**
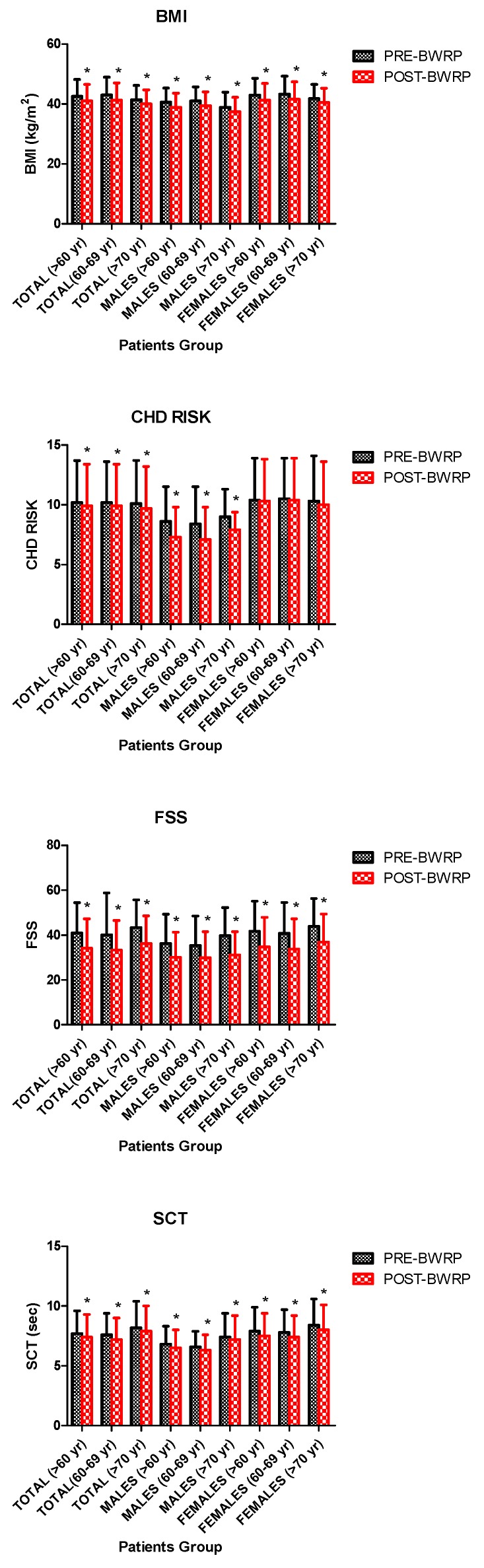
Body mass index (BMI, top panel), coronary heart disease risk (CHD RISK, top middle panel), fatigue severity scale (FSS) score (bottom middle panel) and stair climbing test (SCT) time (bottom panel) before and after a three-week body weight reduction program (BWRP) in elderly obese females and males. Data are expressed as mean ± SD. The values corresponding to each gender- and age-related subgroup (total, females/males, >60 years, 60–69 years and >70 years) are reported. * *p* < 0.01 compared to before body weight reduction program (BWRP).

**Figure 3:**
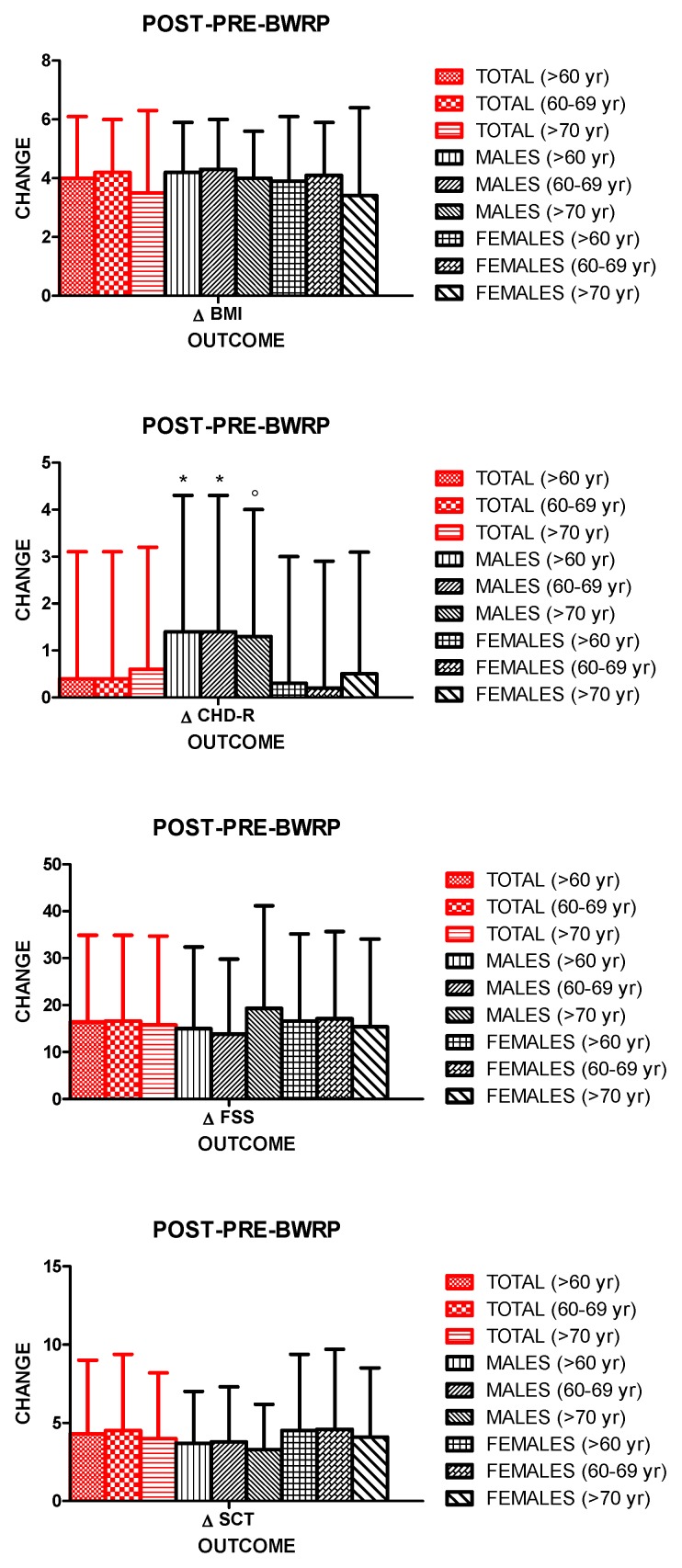
Changes of body mass index (ΔBMI, top panel), coronary heart disease risk (ΔCHD-R, top middle panel), fatigue severity scale (ΔFSS) score (bottom middle panel) and stair climbing test (ΔSCT) time (bottom panel) before and after a three-week body weight reduction program (POST-PRE-BWRP) in elderly obese females and males. Data are expressed as mean ± SD. The values corresponding to each gender- and age-related subgroup (total, females/males, >60 years, 60–69 years and >70 years) are reported. * *p* < 0.01 compared to the corresponding female subgroup; ° *p* < 0.01 compared to females aged 60–69 years.

BWRP significantly reduced BW and BMI when considering all data, female and male groups and age- and gender-specific subgroups (*p* < 0.01) (Table 1; Figure 2). No significant differences in ΔBW (%) and ΔBMI (%) were observed among all subgroups, indicating a similar effectiveness of the intervention irrespectively from gender (females/males) and age (>60 years, 60–69 years and >70 years) (Table 1; Figure 3). In particular, a ΔBMI of 4.0 ± 2.1% was found for all data (Table 1; Figure 3).

BWRP significantly reduced T-C and HDL-C in all subgroups (all, females/males, >60 years, 60–69 years and >70 years) (*p* < 0.01) (Table 2). Glucose was significantly reduced by BWRP when comparing each subgroup before and after the intervention (*p* < 0.01) (Table 2). No significant differences in ΔT-C, ΔHDL-C and ΔGLU (%) were detected among all subgroups (Table 2). In particular, a ΔT-C, ΔHDL-C and ΔGLU of 13.7 ± 14.3%, 14.8 ± 13.7% and 10.6 ± 14.5%, respectively, were found for all data (Table 2).

SBP and DBP were significantly reduced at the end of BWRP in all subgroups (*p* < 0.01), with the exception of DBP in males aged >70 years (Table 2). No significant differences in ΔSBP and ΔDBP (%) were found among all subgroups (Table 2). In particular, a ΔSBP and ΔDBP of 5.9 ± 9.9% and 3.2 ± 11.7%, respectively, were found for all data (Table 2).

Due to the favorable effect of BWRP on BMI, T-C and BP, a significant decrease in CHD-R was observed at the end of the intervention in all subgroups (*p* < 0.01), with the exception of females aged >60 years, 60–69 years and >70 years (Table 3; Figure 2). Noteworthy, when pooling data from males and females, due to the positive contribution of males aged >60 years, whose CHD-R significantly decreased from 8.6 ± 2.9 to 7.3 ± 2.5, the pre-post-BWRP difference of CHD-R was significant in the two genders considered together (aged >60 years, 60–69 years and >70 years) (*p* < 0.01) (Table 3; Figure 2). There were no significant differences in ΔCHD-R (absolute value) among male or female subgroups irrespectively from age (>60 years, 60–69 years and >70 years); anyway, males aged >60 years or 60–69 years had a higher ΔCHD-R than that in the corresponding female subgroup (*p* < 0.01); furthermore, the ΔCHD-R in males aged >70 years was significantly higher than that in females aged 60–69 years (*p* < 0.01), but not >70 years (Table 1; Figure 3). When pooling data from females and males, ΔCHD-R was 0.4 ± 2.7.

BWRP significantly reduced FSS score and SCT time in all subgroups (all, females/males, >60 years, 60–69 years and >70 years) (*p* < 0.01) (Table 3; Figure 2). There were no significant differences in the ΔFSS score and ΔSCT time (%) among all subgroups (Table 3; Figure 3). In particular, a ΔFSS score and ΔSCT time of 16.4 ± 18.5% and 4.3 ± 4.7%, respectively, were found for all data (Table 3; Figure 3).

**Figure 4:**
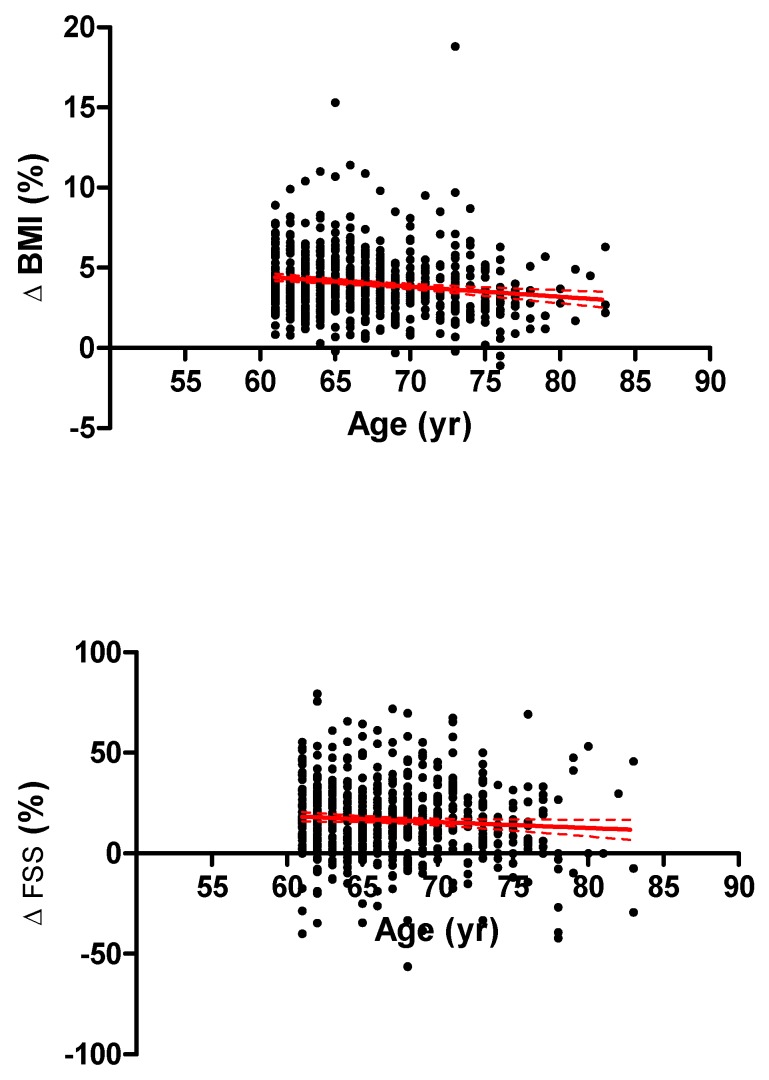
Correlations of age with changes in body mass index (ΔBMI, top panel) or fatigue severity scale (ΔFSS,) score (bottom panel) before and after a three-week body weight reduction program (POST-PRE-BWRP) in elderly obese females and males. The results of the linear regression analysis are reported (i.e., the best fitted line for all data together with 95% confidence interval).

Among all possible correlations, the most relevant were those between ΔBMI (%) and age (*r* = −0.153, *p* < 0.01) and between ΔFSS score (%) and age (*r* = −0.0763, *p* < 0.05) (Figure 4).

## 4. Discussion

The main findings of the present study, carried out in a large cohort of elderly obese females and males, undergoing a three-week multidisciplinary BWRP, including hypocaloric diet, physical activity and psychological counseling, can be summarized in the following key points: (1) BWRP is capable of reducing BW and BMI in an obese geriatric population, including patients aged >70 years, the effects being similar to those obtained in young obese adults (a ΔBMI of about 4%) [21,27,31,32]; (2) BWRP improves some cardiovascular risk factors, such as T-C, glucose, SBP and DBP, with the ensuing reduction of CHD-R (a ΔCHD-R of about 0.4 points), an effect more evident in all male subgroups irrespectively from age; (3) BWRP reduces the FSS score and SCT time, indicating beneficial effects on fatigue in daily activities and motor performance, which are requisites to obtain a better functional autonomy in the later life [33]; (4) an age-dependent reduction in ΔBMI and ΔFSS score is present, the decline between 60 and 80 years being however relatively modest, indicating that BWRP is able to produce beneficial effects even in advanced age.

As stated in recent recommendations by the most important scientific societies in clinical nutrition, geriatrics and obesiology [9], any BWRP administered in elderly obese patients, in addition to reduce obesity-associated medical complications, ought to focus on improving physical function and quality of life.

Reportedly, fatigue is a highly prevalent symptom in obese subjects irrespectively from age, but the prevalence further increases in the geriatric population due to other comorbidities such as osteoarthritis and sarcopenia, which generally are not present in young subjects [24,34]. An obese patient complaining of fatigue can be trapped in a vicious circle because fatigue reduces physical activity and a sedentary state promotes weight gain, with a negative impact on the quality of life [35,36].

As shown in the present study, BWRP significantly reduced both BW and FSS score in all subgroups of patients (male/female or >60 years, 60–69 years and >70 years); nevertheless, no correlation was found between the ΔBMI and ΔFSS score. These findings seem to suggest that the beneficial effects of BWRP on fatigue are not uniquely a consequence of the weight loss and the improved physical agility per se, but also due to a perceived psychological well being [37]. In this context, it is noteworthy to recall the mood-elevating effect of exercise, even when practiced moderately, which can modulate monoaminergic neurotransmission, which governs physical symptoms, including fatigue [38].

SCT is a validated and simple test to assess the maximally attainable lower limbs muscle power output [22,23]. In the present study, the SCT time was shown to improve after BWRP. The relevance of this finding relies on the demographic characteristics of the recruited population, consisting in elderly obese subjects, for whom execution of the test may be more difficult for many age- and not only BW-related reasons in comparison with a younger group [33]. Different factors (i.e., reduction of BW, shift in the balance between parasympathetic and sympathetic activities, central and peripheral adaptations with more efficient muscle contractions and a more favorable left shift of the muscle power-velocity curve, acquisition of motor skillfulness and coordination during the repeated trials, increased self-esteem and motivation) might be invoked, alone or in combination, to explain the positive effects of BMRP on SCT-related motor performance [22,23]. Since a high level of physical activity has been shown to increase the odds of a healthy aging and protect the “frail” elderly patient independently from any effect on BW [39], any effort should be made to engage elderly obese subjects in a BWRP, appropriately tailored to patient’s clinical needs.

The advantage of a (even partially) restored physical agility in an elderly obese subject undergoing a BWRP might overcome the cardiometabolic benefits (see below) or contrast the so-called “obesity paradox”, a debated issue for which there would be a survival advantage in overweight/obese patients, calling into question the importance of weight loss particularly in geriatric population [19].

The present study confirms the effectiveness of our BWRP to reduce the CHD-R in elderly obese patients [40]. Though the duration of the BWRP was limited (only three weeks), the cardiometabolic benefits were relevant as shown by a ΔCHD-R of about 0.4 points, which might correspond to a decreased (not uniquely cardiovascular) mortality over a long time interval, particularly in the subjects aged 60–69 years. Though the imposition of a healthy life style is fundamental to maintain the cardiometabolic benefits produced by a (previous) BWRP over time [41], epidemiological studies in a large geriatric population are mandatory to detract the theory of the “obesity paradox”, including the elimination of methodological biases such as elderly obese patients for whom a catabolic state due to a BWPR would represent a precipitating factor of other pre-existing critical diseases (e.g., oncological or cardiological conditions) [42].

In the present study, ΔCHD-R was higher in males than females and similar among all male subgroups (>60 years, 60–69 years and >70 years); furthermore, BWRP did not significantly reduce CHD-R in female subgroups. These findings are not surprising. In fact, as shown by other studies [43], there is a dramatic increase of CHD-R in females during the menopausal transition, for whom the well-known cardioprotective effects of (endogenous) estrogens are missing [44].

Nevertheless, due to the multisystemic benefits that are produced by a BWRP in both males and females, post-menopausal women, who epidemiologically have a longer life expectancy than men [45] and have a higher risk of weight gain because of the hypoestrogenism [5], should be encouraged to take part in any BWRP to obtain other benefits, e.g., increased motor performance.

Though statistically significant, the correlations of age with the ΔBMI or ΔFSS score do not represent a valid reason to exclude the very elderly subject from a BWRP. In fact, the age-dependent decline of BWRP effectiveness appears to be modest and, despite this fact, as reported above, the BWRP-induced benefits are evident in all subgroups, including subjects aged >70 years. In our opinion, this age-dependent hyporesponsiveness should be further investigated to better optimize the BWRP for very elderly subjects, who could have different clinical needs [7,8,9,10,14].

In conclusion, though only a limited number of outcomes was investigated, the present study shows that a 4% BW reduction following a multidisciplinary BWRP in severely elderly obese patients is associated with positive multisystemic effects, particularly, muscle-skeletal and cardiometabolic benefits, which can favorably influence their general well-being and improve the autonomy level in performing the more common daily activities. The maintenance of healthy life styles, including controlled food intake and regular physical activity, after a period of metabolic rehabilitation (i.e., BWRP), which can be repeated and/or personalized when needed, is strongly recommended in all elderly obese patients to improve their clinical condition and autonomy level [41].

## Figures and Tables

**Table 1:** Demographic and anthropometric characteristics of the study population (before and after BWRP).

		Total			Males			Females	
Parameter	>60 Year	60–69 Year	>70 Year	>60 Year	60–69 Year	>70 Year	>60 Year	60–69 Year	>70 Year
									
No.	684	500	184	92	69	23	592	432	160
Age (year)	67.7 ± 4.7	64.7 ± 2.6	73.4 ± 3.1	67.0 ± 4.8	64.7 ± 2.5	74.1 ± 2.6	67.1 ± 4.7	64.7 ± 2.6	73.4 ± 3.1
WHR	1.0 ± 0.1	0.9 ± 0.1	1.0 ± 0.1	1.0 ± 0.1 ^a^	1.0 ± 0.1 ^a^	1.0 ± 0.1 ^a^	0.9 ± 0.1	0.9 ± 0.1	0.9 ± 0.1
Height (m)	1.6 ± 0.1	1.6 ± 0.1	1.6 ± 0.1	1.7 ± 0.1 ^a^	1.7 ± 0.1 ^a^	1.6 ± 0.1 ^a^	1.5 ± 0.1	1.5 ± 0.1	1.5 ± 0.1
									
PRE-BW (kg)	104.3 ± 15.7	106.0 ± 16.4	99.6 ± 12.3	114.8 ± 16.8 ^a^	118.1 ± 16.7 ^a^	104.9 ± 12.6 ^a,b^	102.6 ± 14.9	104.0 ± 15.5	98.8 ± 12.1 ^b^
POST-BW (kg)	100.3 ± 15.2 ^c^	101.8 ± 15.9 ^c^	96.4 ± 12.2 ^c^	110.1 ± 16.3 ^c^	113.3 ± 16.3 ^c^	98.8 ± 14.4 ^c^	98.8 ± 14.4 ^c^	99.9 ± 15.0 ^c^	95.7 ± 12.0 ^c^
ΔBW (%)	4.7 ± 9.2	4.8 ± 8.7	4.4 ± 10.3	5.3 ± 10.1	5.7 ± 11.6	4.0 ± 1.5	4.6 ± 9.0	4.6 ± 8.1	4.4 ± 11.1
									
PRE-BMI (kg/m^2^)	42.6 ± 5.6	43.0 ± 5.9	41.4 ± 4.8 ^d^	40.6 ± 4.8 ^e^	41.1 ± 4.6 ^e^	38.9 ± 5.0 ^e^	42.9 ± 5.7	43.3 ± 6.0	41.8 ± 4.7 ^f^
POST-BMI (kg/m^2^)	41.0 ± 5.5 ^c^	41.3 ± 5.7 ^c^	40.0 ± 4.8 ^c^	38.9 ± 4.7 ^c^	39.4 ± 4.6 ^c^	37.4 ± 4.8 ^c^	41.3 ± 5.6 ^c^	41.6 ± 5.8 ^c^	40.5 ± 4.7 ^c^
ΔBMI (%)	4.0 ± 2.1	4.2 ± 1.8	3.5 ± 2.8	4.2 ± 1.7	4.3 ± 1.7	4.0 ± 1.6	3.9 ± 2.2	4.1 ± 1.8	3.4 ± 3.0
									

Values are expressed as mean ± SD. ^a^ *p* < 0.01 compared to the corresponding female subgroup of the same age; ^b^ *p* < 0.01 compared to the subgroups of the same gender, aged >60 years and 60–69 years; ^c^ *p* < 0.01 compared to the corresponding subgroup before BWRP; ^d^ *p* < 0.01 compared to the subgroups aged >60 years and 60–69 years, including both females and males (i.e., total); ^e^ *p* < 0.01 compared to the corresponding female subgroup of the same age range; ^f^ *p* < 0.01 compared to the subgroup of the same gender, aged 60–69 years. WHR, waist to hip circumference ratio; BW, body weight; BMI, body mass index; BWRP, body weight reduction program.

**Table 2:** Biochemical and cardiovascular parameters of the study population (before and after BWRP).

		Total			Males			Females	
Parameter	>60 Year	60–69 Year	>70 Year	>60 Year	60–69 Year	>70 Year	>60 Year	60–69 Year	>70 Year
									
PRE-GLU (mg/dL)	107.3 ± 33.9	108.3 ± 35.4	104.5 ± 29.0	105.4 ± 28.0	108.4 ± 30.3	96.1 ± 16.7	107.6 ± 34.7	108.2 ± 36.2	105.8 ± 30.3
POST-GLU (mg/dL)	88.0 ± 14.4 ^a^	87.8 ± 14.9 ^a^	88.5 ± 12.8 ^a^	86.1 ± 12.8 ^a^	86.2 ± 13.0 ^a^	85.8 ± 12.6 ^a^	88.5 ± 14.8 ^a^	88.2 ± 15.4 ^a^	89.3 ± 13.0 ^a^
ΔGLU (%)	10.6 ± 14.5	10.9 ± 14.7	9.6 ± 13.9	11.7 ± 13.8	11.2 ± 15.6	12.9 ± 8.0	10.2 ± 14.8	10.8 ± 14.5	8.5 ± 15.6
									
PRE-T-C (mg/dL)	199.0 ± 38.8	199.2 ± 39.8	198.4 ± 36.1	193.7 ± 36.1	193.6 ± 36.0	194.0 ± 37.3	199.8 ± 39.2	200.1 ± 40.3	199.0 ± 36.1
POST-T-C (mg/dL)	169.7 ± 35.9 ^a^	170.0 ± 36.2 ^a^	168.8 ± 34.8 ^a^	155.9 ± 36.8 ^a^	156.4 ± 39.4 ^a^	154.6 ± 28.0 ^a^	171.8 ± 35.3 ^a^	172.2 ± 35.3 ^a^	170.6 ± 35.3 ^a^
ΔT-C (%)	13.7 ± 14.3	13.5 ± 14.0	14.3 ± 15.1	17.9 ± 16.7	17.8 ± 17.9	18.2 ± 12.6	13.1 ± 13.8	12.8 ± 13.2	13.8 ± 15.4
									
PRE-HDL-C (mg/dL)	50.9 ± 13.5	50.5 ± 13.9	51.7 ± 12.1 ^b^	40.9 ± 9.2 ^c^	40.0 ± 9.0 ^c^	43.8 ± 9.6 ^c^	52.4 ± 13.4	52.2 ± 13.9	52.9 ± 12.1
POST-HDL-C (mg/dL)	42.7 ± 10.3 ^a^	42.6 ± 10.6 ^a^	43.0 ± 9.3 ^a^	35.6 ± 7.9 ^a^	34.8 ± 7.8 ^a^	38.1 ± 7.8 ^a^	43.8 ± 10.2 ^a^	43.8 ± 10.5 ^a^	43.8 ± 9.3 ^a^
ΔHDL-C (%)	14.8 ± 13.7	14.3 ± 13.2	16.0 ± 15.0	11.0 ± 13.3	10.9 ± 13.8	11.1 ± 11.8	15.4 ± 13.7	14.9 ± 13.0	16.7 ± 15.3
									
PRE-DBP (mmHg)	77.6 ± 8.2	77.8 ± 7.9	76.9 ± 8.8	76.8 ± 9.5	78.0 ± 9.0	73.3 ± 10.5	77.7 ± 7.9	77.8 ± 7.7	77.4 ± 8.4
POST-DBP (mmHg)	74.6 ± 6.7 ^a^	74.6 ± 7.0 ^a^	74.4 ± 5.9 ^a^	74.7 ± 7.5 ^a^	74.9 ± 7.9 ^a^	74.3 ± 6.2	74.5 ± 6.6 ^a^	74.5 ± 6.8 ^a^	74.5 ± 5.9 ^a^
ΔDBP (%)	3.2 ± 11.7	3.5 ± 10.8	2.6 ± 13.8	2.7 ± 16.8	3.3 ± 10.7	1.0 ± 28.7	3.3 ± 10.7	3.5 ± 10.8	2.9 ± 10.5
									
PRE-SBP (mmHg)	132.6 ± 14.6	132.3 ± 14.4	133.4 ± 15.4	131.5 ± 14.5	32.4 ± 14.0	128.6 ± 16.0	132.8 ± 14.7	132.2 ± 14.5	134.2 ± 15.1
POST-SBP (mmHg)	123.8 ± 10.2 ^a^	123.7 ± 10.3 ^a^	124.0 ± 9.9 ^a^	123.9 ± 11.1 ^a^	124.0 ± 11.4 ^a^	123.6 ± 10.4 ^a^	123.8 ± 10.0 ^a^	123.7 ± 10.1 ^a^	124.1 ± 9.9 ^a^
ΔSBP (%)	5.9 ± 9.9	5.7 ± 10.2	6.4 ± 9.1	5.0 ± 9.7	5.4 ± 10.0	3.7 ± 8.6	6.0 ± 9.9	5.7 ± 10.2	6.0 ± 9.1
									

Values are expressed as mean ± SD. ^a^ *p* < 0.01 compared to the corresponding subgroup before BWRP; ^b^ *p* < 0.01 compared to the subgroup aged 60–69 years, including both females and males (i.e., total); ^c^ *p* < 0.01 compared to the corresponding female subgroup of the same age. BWRP, body weight reduction program; GLU, glucose; T-C, total cholesterol; HDL-C, HDL cholesterol; DBP, diastolic blood pressure; SBP, systolic blood pressure.

**Table 3:** CHD-R, FSS score and SCT time in the study population (before and after BWRP).

		Total			Males			Females	
Outcome	>60 Year	60–69 Year	>70 Year	>60 Year	60–69 Year	>70 Year	>60 Year	60–69 Year	>70 Year
									
PRE-CHD-R (points)	10.2 ± 3.5	10.2 ± 3.4	10.1 ± 3.6 ^a^	8.6 ± 2.9 ^b^	8.4 ± 3.1 ^b^	9.0 ± 2.3	10.4 ± 3.5	10.5 ± 3.4	10.3 ± 3.8 ^c^
POST-CHD-R (points)	9.9 ± 3.5 ^d^	9.9 ± 3.5 ^d^	9.7 ± 3.5 ^d^	7.3 ± 2.5 ^d^	7.1 ± 2.7 ^d^	7.9 ± 1.5 ^d^	10.3 ± 3.5	10.4 ± 3.5	10.0 ± 3.6
ΔCHD-R (points)	0.4 ± 2.7	0.4 ± 2.7	0.6 ± 2.6	1.4 ± 2.9 ^b^	1.4 ± 2.9 ^b^	1.3 ± 2.7 ^c^	0.3 ± 2.7	0.2 ± 2.7	0.5 ± 2.6
									
PRE-FSS score (points)	40.9 ± 13.5	40.1 ± 13.7	43.3 ± 12.4	36.2 ± 13.1 ^b^	35.2 ± 13.2 ^b^	39.7 ± 12.6	41.7 ± 13.4	40.8 ± 13.7	43.9 ± 12.3
POST-FSS score (points)	34.1 ± 13.1 ^d^	33.3 ± 13.2 ^d^	36.2 ± 12.4 ^d^	30.1 ± 11.2 ^d^	29.9 ± 11.5 ^d^	31.1 ± 10.3 ^d^	34.7 ± 13.2 ^d^	33.8 ± 13.4 ^d^	36.9 ± 12.5 ^d^
ΔFSS score (%)	16.4 ± 18.5	16.6 ± 18.3	15.8 ± 18.9	15.0 ± 17.4	13.8 ± 16.0	19.3 ± 21.9	16.6 ± 18.6	17.1 ± 18.6	15.4 ± 18.7
									
PRE-SCT time (sec)	7.7 ± 1.9	7.6 ± 1.8	8.2 ± 2.2	6.8 ± 1.5 ^b^	6.6 ± 1.3 ^b^	7.4 ± 2.0	7.9 ± 2.0	7.8 ± 1.9	8.4 ± 2.2
POST-SCT time (sec)	7.4 ± 1.9 ^d^	7.2 ± 1.8 ^d^	7.9 ± 2.1 ^d^	6.5 ± 1.5 ^d^	6.3 ± 1.3 ^d^	7.2 ± 2.0 ^d^	7.5 ± 1.9 ^d^	7.4 ± 1.8 ^d^	8.0 ± 2.1 ^d^
ΔSCT time (%)	4.3 ± 4.7	4.5 ± 4.9	4.0 ± 4.2	3.7 ± 3.3	3.8 ± 3.5	3.3 ± 2.9	4.5 ± 4.9	4.6 ± 5.1	4.1 ± 4.4
									

Values are expressed as mean ± SD. ^a^ *p* < 0.01 compared to the subgroup aged 60–69 years, including both females and males (i.e., total); ^b^ *p* < 0.01 compared to the corresponding female subgroup of the same age range; ^c^ *p* < 0.01 compared to the female subgroup aged 60–69 years. ^d^ *p* < 0.01 compared to the corresponding subgroup before BWRP; BWRP, body weight reduction program; CHD-R, coronary heart disease risk; FSS, fatigue severity scale; SCT, stair climbing test.

## Data Availability

The datasets used and/or analyzed in the present study are available from the corresponding author on reasonable request.
